# Neurovascular control during exercise in acute coronary syndrome patients with Gln27Glu polymorphism of β_2_-adrenergic receptor

**DOI:** 10.1371/journal.pone.0173061

**Published:** 2017-02-24

**Authors:** Larissa Ferreira-Santos, Daniel G. Martinez, José Carlos Nicolau, Humberto G. Moreira, Maria Janieire Alves, Alexandre C. Pereira, Ivani C. Trombetta, Carlos Eduardo Negrão, Maria Urbana P. B. Rondon

**Affiliations:** 1 Heart Institute (InCor-HCFMUSP), University of São Paulo Medical School, São Paulo, Brazil; 2 School of Physical Education and Sport, University of São Paulo, São Paulo, Brazil; 3 Faculty of Physical Education and Sports, Federal University of Juiz de Fora, Juiz de Fora, Brazil; 4 Universidade Nove de Julho (UNINOVE), São Paulo, Brazil; Temple University, UNITED STATES

## Abstract

**Background:**

Gln27Glu (rs1042714) polymorphism of the β_2_-adrenergic receptor (ADRB2) has been association with cardiovascular functionality in healthy subjects. However, it is unknown whether the presence of the ADRB2 Gln27Glu polymorphism influences neurovascular responses during exercise in patients with acute coronary syndromes (ACS). We tested the hypothesis that patients with ACS homozygous for the Gln allele would have increased muscle sympathetic nerve activity (MSNA) responses and decreased forearm vascular conductance (FVC) responses during exercise compared with patients carrying the Glu allele (Gln27Glu and Glu27Glu). In addition, exercise training would restore these responses in Gln27Gln patients.

**Methods and results:**

Thirty-days after an ischemic event, 61 patients with ACS without ventricular dysfunction were divided into 2 groups: (1) Gln27Gln (n = 35, 53±1years) and (2) Gln27Glu+Glu27Glu (n = 26, 52±2years). MSNA was directly measured using the microneurography technique, blood pressure (BP) was measured with an automatic oscillometric device, and blood flow was measured using venous occlusion plethysmography. MSNA, mean BP, and FVC were evaluated at rest and during a 3-min handgrip exercise. The MSNA (*P* = 0.02) and mean BP (*P* = 0.04) responses during exercise were higher in the Gln27Gln patients compared with that in the Gln27Glu+Glu27Glu patients. No differences were found in FVC. Two months of exercise training significantly decreased the MSNA levels at baseline (*P* = 0.001) and in their response during exercise (*P* = 0.02) in Gln27Gln patients, but caused no changes in Gln27Glu+Glu27Glu patients. Exercise training increased FVC responses in Gln27Glu+Glu27Glu patients (*P* = 0.03), but not in Gln27Gln patients.

**Conclusion:**

The exaggerated MSNA and mean BP responses during exercise suggest an increased cardiovascular risk in patients with ACS and Gln27Gln polymorphism. Exercise training emerges as an important strategy for restoring this reflex control. Gln27Glu polymorphism of ADRB2 influences exercise-induced vascular adaptation in patients with ACS.

## Introduction

Acute coronary syndrome (ACS) is associated with autonomic and hemodynamic alterations [1–3]. Patients with myocardial infarction have exaggerated muscle sympathetic nerve activity (MSNA) levels and reduced forearm blood flow (FBF) responses during exercise compared with healthy individuals [2].

Accumulated evidence shows that the β_2_-adrenergic receptor (ADRB2) plays an important role in neurovascular regulation [4–6]. Intra-arterial infusion of ADRB2 agonist provokes a significant increase in FBF in humans [4,7]. More recently, it has been documented that polymorphism in the NH_2_-terminus ADRB2 in codon 27 at position 79 caused by exchange of the nitrogenous base guanine for cytosine, changing the amino acid glutamic acid (Glu) for glutamin (Gln) [8–10] influences vascular responses during physiological maneuvers. Individuals carrying Glu27Glu have augmented muscle vasodilation during exercise and mental stress [11,12]. Similarly, increased responses to intra-arterial infusion of isoproterenol have been reported in individuals carrying Glu27Glu [13]. In contrast, the presence of Glu27Glu polymorphism of the ADRB2 attenuates agonist-induced vascular responses [14].

Previous observational studies show that the frequency of the Gln27Gln genotype is greater in patients with myocardial infarction than in healthy subjects [15,16]. In addition, patients with ACS who carry the Gln allele have a higher overall mortality rate compared with those who carry the Glu allele [17]. This poor prognosis is suggestive of worsening vascular function in patients who carry the NH_2_-terminus ADRB2 polymorphism. In the present study, we describe neurovascular control during exercise in patients with ACS who carry the ADRB2 polymorphism.

Exercise training has been shown to cause remarkable neurovascular adaptations in humans with cardiovascular disease. In patients after myocardial infarction, exercise training reduces MSNA, which seems to be associated with improvement in arterial baroreflex control [1]. These benefits of exercise training can be extended to patients with chronic heart failure. This nonpharmacological strategy significantly reduces MSNA and muscle vasoconstriction in patients suffering from systolic cardiac dysfunction [18–21]. The effects of exercise training have also been reported during exercise [22]. However, the effects of exercise training on neurovascular control in patients with ACS and the ADRB2 polymorphism are virtually unknown. In the present study, we describe the neurovascular adaptation provoked by exercise training in patients with ACS who are carriers of the ADRB2 polymorphism.

We tested the following hypotheses: (1) Patients with ACS homozygous for the Gln allele of the ADRB2 polymorphism would have increased MSNA and decreased FBF responses during exercise compared with patients carrying the Glu allele; and (2) exercise training would restore neurovascular control during exercise in patients with ACS who carry the Gln allele.

## Materials and methods

### Subjects

The study was approved by the Scientific Commission of the Heart Institute (InCor) of the University of São Paulo Medical School (#SDC 2326/03/120) and by the Ethics Committee of the Clinical Hospital of the University of São Paulo Medical School (#980/03). All participants provided written informed consent before inclusion in the study, and some of the patients were involved in a previous study [1]. One month after the ischemic event, 61 patients with ACS without left ventricular dysfunction (ejection fraction ≥ 45%) were included in the study. Patients were genotyped for the ADRB2 Gln27Glu (rs1042714) polymorphism and then were divided into 2 groups according to their genotypes: (1) Gln27Gln (*n* = 35) and (2) Gln27Glu + Glu27Glu (*n* = 26). Of these, 29 patients agreed to participate in an exercise training protocol for a period of 8 weeks, but only 25 patients completed the experimental protocol (Gln27Gln, *n* = 17; Gln27Glu + Glu27Glu, *n* = 08).

### Genotyping protocol

Genomic DNA was extracted from leukocytes in samples of venous whole blood. Genotypes were identified by “polymerase chain reaction” (PCR), as previously described [23]. The analyses of amplifications of PCR of the segments the ADRB2 gene was performed by automatic apparatus (Perkin Elmer Corporation, Foster City, CA, USA).

### Cardiopulmonary exercise testing

Maximal exercise capacity was determined during a maximal progressive exercise test on a cycle ergometer (Medifit 400 L, Medical Fitness Equipment, Maarn, The Netherlands), using a ramp protocol with work rate increments of 5–10W every minute until exhaustion. Oxygen uptake (VO_2_) and carbon dioxide production were determined by means of gas exchange on a breath-by-breath basis in a computerized system (CAD/Net 2001, Medical Graphics Corporation, St. Paul, Minnesota, USA). Maximal exercise capacity was determined by the VO_2_ measured at peak of exercise (VO_2_ peak). Anaerobic threshold was identified at the breakpoint between the increase in the carbon dioxide output and VO_2_ (V slope) or at the point in which the ventilatory equivalent for oxygen and end-tidal oxygen partial pressure curves reached their respective minimum values and began to rise. The respiratory compensation point was determined to occur at the point at which the ventilatory equivalent for carbon dioxide ratio inverts its trend toward an initial decrease and systematically increases and when end-tidal carbon dioxide partial pressure reaches a maximum and begins to decrease [24–26].

### Muscle sympathetic nerve activity

Muscle sympathetic nerve activity (MSNA) was measured directly from the peroneal nerve using the microneurography technique, as previously described [27,28]. In brief, multiunit postganglionic muscle sympathetic nerve recordings were made using a tungsten microelectrode (tip diameter 5 to 15 μm). Signals were amplified by a factor of 50,000 to 100,000 and band-passed filtered (700 to 2,000 Hz). For recordings and analysis, nerve activity was rectified and integrated (time constant: 0.1 s) to obtain a mean voltage display. MSNA was expressed as burst frequency (bursts/min), and burst incidence (bursts/100 heart beats).

### Forearm blood flow

Forearm blood flow (FBF) was measured by venous occlusion plethysmography as previously described [11]. Sphygmomanometer cuffs were placed around the nondominant wrist and upper arm, and a mercury-filled silastic tube attached to a transducer was placed around the forearm and connected to a plethysmograph (Hokanson, Bellevue, Washington, USA). FBF (mL/min/100mL) was determined based on a minimum of 3–4 separate readings per minute. Forearm vascular conductance (FVC) was calculated by dividing FBF by the mean blood pressure times 100 and expressed in arbitrary units.

### Handgrip exercise

After the maximal voluntary contraction was measured (average of 3 repetitions), the isometric handgrip exercise was performed at 30% of maximal voluntary contraction using a handgrip dynamometer.

### Exercise training program

One month after an ischemic event, patients underwent supervised exercise training at the Heart Institute (InCor) of the University of São Paulo Medical School. The 8-week training program consisted of three 60-minute exercise sessions per week. Each exercise session consisted of 5 minutes of stretching, 40 minutes of cycling on an ergometer bicycle, 10 minutes of local strengthening, followed by 5 minutes of cool down with stretching exercises as previously described [1]. The intensity of the exercise was established at a heart rate corresponding to the anaerobic threshold obtained in the cardiopulmonary exercise test. The aerobic exercise was monitored by ECG during all sessions.

### Other measurements

Blood pressure (BP) was monitored noninvasively and intermittently from an automatic and oscillometric cuff (Dixtal, DX 2710; Manaus, Brazil). Heart rate (HR) was monitored continuously through lead II of the ECG.

### Experimental protocol

After patient instrumentation and an adequate nerve recording was obtained, the evaluation of MSNA, FBF, FVC, BP, and HR were performed during 3 minutes at rest and 3 minutes of handgrip exercise at 30% of MVC. All evaluations were performed one month after the ischemic event, and, for those patients undergoing the exercise training program, the same evaluations were repeated after 8 weeks of intervention. All variables were recorded on a computer sampling frequency of 500 Hz and analyzed using Windaq software. All studies were performed at approximately 8:00 AM, with the subjects lying supine in a quiet, air-conditioned room (21°C to 23°C).

### Statistical analysis

The data are presented as mean±SE. Kolmogorov-Smirnov test was used to assess the normality of distribution of each variable studied. Chi-square (χ^2^) test was used to compare categorical data differences. Baseline physical and clinical characteristics and the responses (absolute changes) between groups during isometric exercise were tested using the unpaired Student *t*-test. Differences in the groups before and after exercise training were tested by 2-way ANOVA for repeated measures. When a significant difference was found, Scheffé´s post-hoc comparison test was used. Probability values of *P*<0.05 were considered statistically significant.

## Results

### Baseline characteristics

Physical and clinical characteristics and medications used by patients are displayed in Table 1. There were no significant differences in sex, age, weight, height, body mass index, and left ventricular ejection fraction between groups. The Gln27Gln and Gln27Glu+Glu27Glu groups were similar for the diagnosis of unstable angina and non-ST-segment elevation myocardial infarction. Interestingly, the Gln27Gln group had several patients with ST-segment elevation myocardial infarction significantly higher than that in the Gln27Glu+Glu27Glu group (Table 1). The groups were similar regarding the number of ‘stents utilized’ and prescribed medications.

**Table 1:** Physical and clinical characteristics in patients with polymorphisms Gln27Gln and Gln27Glu+Glu27Glu one month after the ischemic event.

	Gln27Gln (n = 35)	Gln27Glu+Glu27Glu (n = 26)	*P* Value
**Physical Characteristics**			
Sex, men (%)	26 (74)	19 (73)	0.92
Age, years	53±1	52±1	0.69
Weight, kg	76.6±2.2	80.5±3.0	0.29
Height, cm	1.67±0.02	1.69±0.02	0.43
BMI, kg/m^2^	27±1	28±1	0.45
LVEF, %	57±1	59±2	0.30
**Clinical Characteristics, n(%)**			
Unstable Angina	4 (11)	4 (15)	0.65
NSTEMI	12 (34)	15 (58)	0.07
STEMI	19 (54)	7 (27)	0.03
Coronary angiography	35 (100)	26 (100)	1.00
1 stent	29 (83)	19 (73)	0.37
2 stents	2 (6)	2 (8)	0.71
3 stents	1 (3)	0 (0)	0.39
**Medications, n(%)**			
β-adrenergic blocker	32 (91)	25 (96)	0.46
ACE/AT1 inhibitor	34 (97)	25 (96)	0.83
Antiplatelet therapy	35 (100)	26 (100)	1.00
Statin	30 (86)	24 (92)	0.42

Values are mean±SE. BMI, body mass index; LVEF, left ventricular ejection fraction; NSTEMI, non-ST-segment elevation myocardial infarction; STEMI, ST-segment elevation myocardial infarction; ACE, angiotensin-converting enzyme; AT, angiotensin.

### Neurovascular responses to exercise

**Fig 1:**
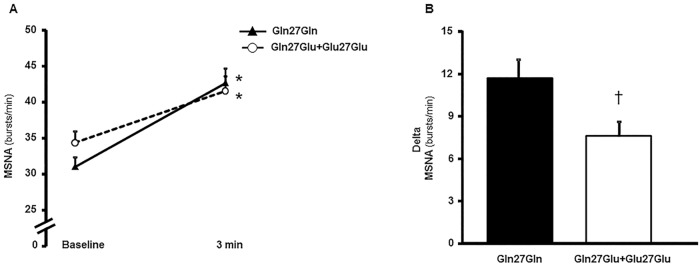
Muscle Sympathetic Nerve Activity (MSNA) at baseline and during handgrip exercise one month after the ischemic event. MSNA in burst frequency (bursts/min) at baseline and during handgrip exercise (A), and the MSNA response (Delta, B) in patients with polymorphisms Gln27Gln and Gln27Glu+Glu27Glu. Note that MSNA increased in both groups during the handgrip exercise, but the MSNA response in the Gln27Gln group is significantly higher compared with that in the Gln27Glu+Glu27Glu group. *P<0.05, difference vs. baseline; ^†^*P*<0.05, difference vs. Gln27Gln.

There were no significant differences between groups for baseline hemodynamic and neurovascular measurements one month after the ischemic event (Table 2). The maximal voluntary contraction was similar in both groups (*P* = 0.78). During the handgrip exercise at 30% of maximal voluntary contraction, MSNA in bursts/min (Fig 1A), mean BP, heart rate (HR), FBF, and FCV increased significantly in both groups (Table 2). Yet, MSNA in bursts/100HB increased from baseline only in the Gln27Gln group (Table 2). However, the responses (delta) of MSNA in bursts/min (*P* = 0.02, Fig 1B) and in bursts/100HB (*P* = 0.02) and mean BP (*P* = 0.04) were higher in the Gln27Gln group compared with the Gln27Glu+Glu27Glu group (Table 2). The HR, FBF, and FVC responses during exercise were similar in both groups (Table 2).

**Table 2:** Hemodynamic and neurovascular characteristics at baseline and during the handgrip exercise in patients with polymorphisms Gln27Gln and Gln27Glu+Glu27Glu one month after the ischemic event.

	Gln27Gln (n = 35)			Gln27Glu+Glu27Glu (n = 26)		
	Baseline	3 min ex	Delta	Baseline	3 min ex	Delta
**MBP** (mmHg)	96±2	120±3*	24±2	101±2	119±2*	18±2^†^
**HR** (beats/min)	54±1	62±2*	8±1	54±2	62±1*	7±1
**MSNA** (bursts/100HB)	59±3	70±3*	11±2	64±3	68±4	4±2^†^
**FBF** (ml/min/100ml)	1.53±0.07	2.07±0.11*	0.54±0.08	1.64±0.11	2.16±0.13*	0.51±0.06
**FVC** (units)	1.63±0.07	1.78±0.10*	0.15±0.08	1.63±0.11	1.79±0.10*	0.16±0.07

Values are mean±SE. MBP, mean blood pressure; HR, heart rate; MSNA, muscle sympathetic nerve activity; FBF, forearm blood flow; FVC, forearm vascular conductance.

*P<0.05 vs. baseline (*2-way ANOVA for repeated measurements*).

^†^*P*<0.05 vs. Gln27Gln (delta analysis, *unpaired t-test)*.

### Exercise training

**Fig 2:**
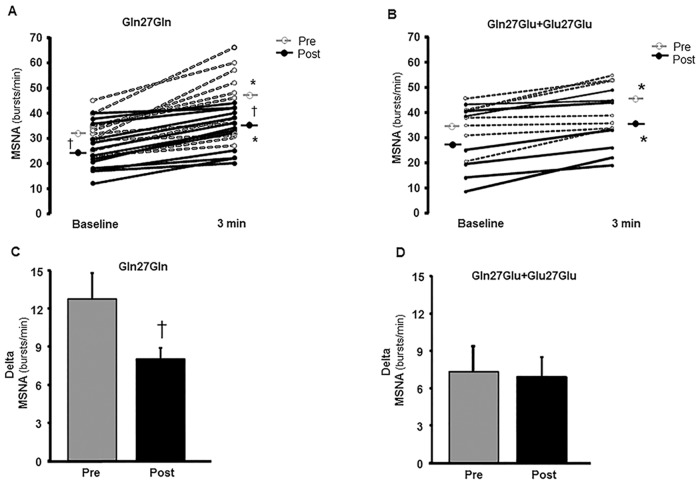
Muscle Sympathetic Nerve Activity (MSNA) pre- and postexercise training period. Individual data for MSNA in burst frequency (bursts/min) at baseline and during the handgrip exercise and the MSNA response (Delta) in patients with polymorphism Gln27Gln (*n* = 17, 2A and 2C, respectively) and Gln27Glu+Glu27Glu (*n* = 08, 2B and 2D, respectively), pre- and postexercise training period. Note that the MSNA significantly decreased after the exercise training period at baseline and during exercise in the Gln27Gln group. *P<0.05, vs. baseline; ^†^*P*<0.05, vs. preintervention.

After exercise training, baseline MSNA in burst frequency and in bursts/100HB decreased in the Gln27Gln group (*P* = 0.01, Fig 2A and *P* = 0.001, Table 3, respectively) and tended to decrease in the Gln27Glu+Glu27Glu group (*P* = 0.052, Fig 2B and *P* = 0.059, Table 3, respectively). Baseline mean BP, HR, FBF, and FVC did not significantly change after exercise training in either group (Table 3).

**Table 3:** Hemodynamic and neurovascular characteristics at baseline and during handgrip exercise in patients with polymorphisms Gln27Gln and Gln27Glu+Glu27Glu before and after the exercise training period.

		Gln27Gln (n = 17)			Gln27Glu+Glu27Glu (n = 08)		
		Baseline	3 min ex	Delta	Baseline	3 min ex	Delta
**MBP** (mmHg)	**Pre**	98±3	122±4*	24±3	100±3	119±3*	19±3
	**Post**	98±2	120±3*	22±3	103±4	115±3*^#^	11±2^†^
**HR** (beats/min)	**Pre**	53±1	60±2*	7±2	55±3	63±3*	7±2
	**Post**	53±2	59±2*	7±1	53±3	58±2*	5±2
**MSNA** (bursts/100HB)	**Pre**	62±4	74±4*	12±2	68±4	75±4*	7±3
	**Post**	47±5^#^	53±4*^#^	5±2^#^	55±6	62±7*	7±3
**FBF** (ml/min/100ml)	**Pre**	1.77±0.10	2.13±0.18*	0.37±0.08	1.64±0.10	2.24±0.17*	0.59±0.11
	**Post**	1.70±0.13	2.19±0.17*	0.50±0.07	1.89±0.24	2.53±0.33*	0.64±0.15
**FVC** (units)	**Pre**	1.78±0.10	1.73±0.13	-0.05±0.08	1.66±0.12	1.89±0.12*	0.23±0.09
	**Post**	1.76±0.12	1.83±0.15	0.07±0.08	1.58±0.20	2.10±0.24*	0.53±0.19^†^

Values are mean±SE. MBP, mean blood pressure; HR, heart rate; MSNA, muscle sympathetic nerve activity; FBF, forearm blood flow; FVC, forearm vascular conductance; Pre, preintervention period; Post, after exercise training period.

^#^ *P*<0.05, within-group comparisons vs. preintervention (*two-way ANOVA for repeated measurements*).

*P<0.05, within-group comparisons vs. baseline.

^†^*P*<0.05, between-group comparisons (delta analysis, *unpaired t-test)*.

Relative to the effect of exercise training during the handgrip exercise at 30% of maximal voluntary contraction, the levels and the response (delta) of MSNA decreased in the Gln27Gln group either in burst frequency (*P* = 0.004, Fig 2A and *P* = 0.04, Fig 2C, respectively) or bursts/100HB (*P*<0.001 and *P* = 0.02, respectively, Table 3). In contrast, exercise training did not significantly change the levels or the response (delta) of MSNA in burst frequency (Fig 2B and 2D, respectively) and in bursts/100HB (Table 3) in the Gln27Glu+Glu27Glu group.

The level and the response (delta) of mean BP during exercise did not significantly change after exercise training in the Gln27Gln group (Table 3). However, in the Gln27Glu+Glu27Glu group, the level of mean BP (*P* = 0.02) decreased, and the response (delta) showed a tendency to decrease (*P* = 0.07) after exercise training (Table 3). HR, FBF, and FVC during exercise did not significantly change after exercise training in either group (Table 3).

**Fig 3:**
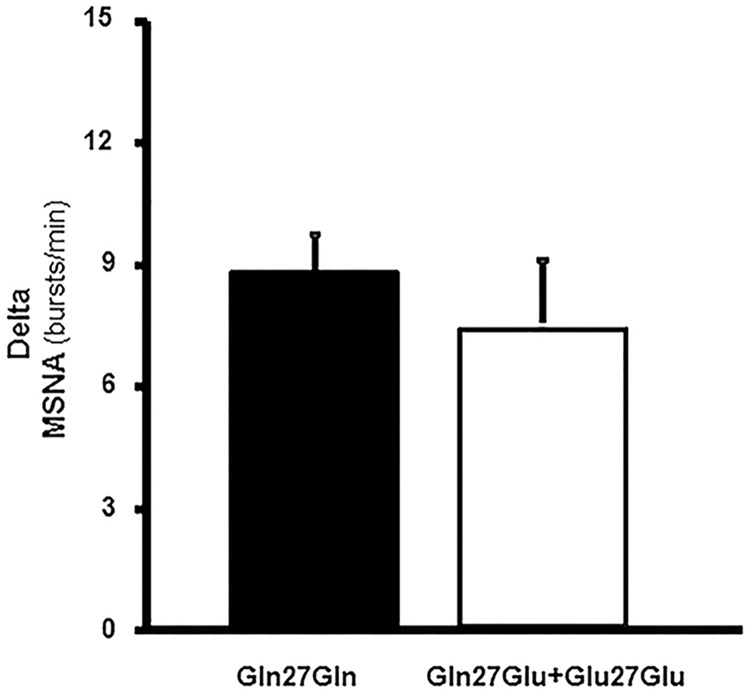
Muscle Sympathetic Nerve Activity (MSNA) response in the postexercise training period. MSNA response (delta) in bursts/min in the postexercise training period in patients with the polymorphism Gln27Gln (*n* = 17) and in the Gln27Glu+Glu27Glu group (*n* = 08). Note that MSNA response was similar in both groups after the exercise training period.

Further analysis between groups in the postexercise training period showed that the initial difference in MSNA response during exercise in burst frequency and bursts/100HB were no longer observed (Fig 3 and Table 3). In addition, in the postexercise training period, the mean BP response during exercise was lower (delta, *P* = 0.01) and the FVC response was higher (delta, *P* = 0.03) in the Gln27Glu+Glu27Glu group compared with that in the Gln27Gln group (Table 3).

## Discussion

The main findings of the present study are that (1) patients with ACS with the Gln27Gln ADRB2 polymorphism have higher responses of MSNA and MBP during exercise compared with patients with Gln27Glu+Glu27Glu polymorphisms; (2) an exercise-based cardiac rehabilitation program results in a decline of MSNA levels at baseline and in their response during a handgrip exercise in patients with the Gln27Gln polymorphism towards the response of patients with Gln27Glu+Glu27Glu polymorphisms; (3) exercise training increases muscle vasodilatation and lowers mean BP responses during handgrip exercises in patients with Gln27Glu+Glu27Glu polymorphisms, but not in patients with the ADRB2 Gln27Gln polymorphism.

### Neurovascular responses during exercise

To the best of our knowledge, this is the first study evaluating the response of MSNA in patients with ACS with the ADRB2 polymorphism. Interestingly, despite optimal medical therapy, patients with the Gln27Gln polymorphism have an exaggerated response of MSNA during exercise one month after an ischemic event compared with their counterparts with the Gln27Glu+Glu27Glu polymorphisms. The sympathetic overactivation during exercise observed in the patients with ACS with the Gln27Gln polymorphism apparently has hemodynamic implications. Blood pressure responses during this physiological condition are higher in these patients. Similar findings have been reported in healthy men who are carriers of the Gln27 allele of the ADRB2. These individuals have higher levels of MSNA compared with subjects homozygous for Glu27 [29]. In addition, this neurovascular marker may contribute to the worse prognosis of patients with Gln27Gln genotype after myocardial infarction [17]. We have previously reported that MSNA is an independent predictor of mortality in patients with chronic heart failure [30].

The mechanism involved in the increased sympathetic nerve activity in Gln27Gln group was out of the scope of our study. However, we can speculate that the desensitization and/or downregulation of the β-adrenergic receptors contribute to this response [9,14,31]. The impairment in functionality of β-adrenergic receptors caused by persistent receptor stimulation due to augmented catecholamine levels leads to a redistribution of the recognition sites for β-adrenergic receptors, decreasing their number in the plasmatic membrane (downregulation) and increasing the internalization of these receptors in the cytosol (desensitization) [32–35]. In fact, the time course for desensitization during the terbutaline infusion protocol was slower in subjects homozygous for Glu27Glu compared with those homozygous for Gln27Gln [14]. On the other hand, we cannot rule out the possibility that ADRB2 polymorphism may affect its sensitivity to beta-blockers and in consequence, to influence MSNA at baseline and during exercise. This issue was out of the scope of the present study. However, we observed that the number of patients taking beta-blockers was similar between groups (Table 1). Besides, further analysis showed that beta-blocker dosage was equal between groups (Gln27Gln: 58±6 mg/day vs. Gln27Glu+Glu27Glu: 60±6 mg/day, P = 0.89). Thus, as previously described, the levels of heart rate were similar between groups. In addition, our data shown that the different responses of MSNA between Gln27Gln and Gln27Glu+Glu27Glu groups were observed in both bursts per minute and in bursts corrected by heart beats. Nevertheless, future investigations should focus on this issue.

In the present study, we observed a similar vasodilatory response between Gln27Gln and Gln27Glu+Glu27Glu groups one month after an ischemic event. This is an intriguing finding, because in a previous study, we found that healthy individuals Glu27Glu homozygous for ADRB2 had higher vasodilatation during exercise and mental stress than individuals Gln27Gln homozygous [11,12]. In addition, Dishy et al. [36] reported that healthy individuals who are carriers of the Glu27Glu polymorphism had higher muscle vasodilatation in response to isoproterenol infusion. This controversy may be explained by the fact that we are dealing with patients with ACS. In fact, we previously documented that patients after acute myocardial infarction had blunted vasodilatory response during exercise [2], and other authors have demonstrated that endothelium-dependent vasodilatory dysfunction is present in these patients [37–39]. It is unlikely that the use of β-blockers explains the difference in muscle vasodilatory responses between Gln27Gln and Gln27Glu+Glu27Glu groups, because 91% of Gln27 patients were using β-blockers, (54% selective and 37% nonselective, data not shown) versus 96% of Gln27Glu+Glu27Glu patients using β-blockers, (77% selective and 19% nonselective, data not shown).

### Effects of exercise training

Exercise training has been shown to provoke a remarkable reduction in resting MSNA levels in patients with myocardial infarction [1]. Moreover, we have previously described that exercise training decreased MSNA levels during exercise in chronic heart failure patients [22]. The present study extends the knowledge that a genetic profile can influence this autonomic regulation. Eight weeks of an exercise-based cardiac rehabilitation program caused a significant reduction in MSNA levels at baseline and in their response during a handgrip exercise in patients with the Gln27Gln polymorphism. There is no definitive explanation for this training adaptation. However, someone could suggest that amelioration in arterial baroreflex control and reduction of chemoreflex hypersensitivity contribute to the reduction in MSNA. It is also possible that the MSNA response during exercise was mediated by an improvement in afferent muscle reflex control, as recently demonstrated [40].

Another interesting finding in our study is the effect of exercise training on vascular function in patients with ACS and the ADRB2 polymorphism. In contrast to Gln27Gln patients, exercise training significantly increased exercise FVC responses in patients with Gln27Glu+Glu27Glu polymorphisms. This is important information. First, it may explain the heterogeneous response to exercise training across individuals with cardiovascular disease. Second, it explains the better prognosis in patients carrying Gln27Glu+Glu27Glu polymorphisms. Our study provides no information regarding the mechanisms underlying the vascular changes in patients with Gln27Glu+Glu27Glu polymorphisms. However, it is consistent with the notion of increased nitric oxide bioavailability and reduced oxidative stress in skeletal muscle consistently shown in previous studies [41–43].

On the basis of our data, someone could argue that exercise training brings more benefits in FBF and FVC increases during exercise in Gln27Glu+Glu27Glu group compared with Gln27Gln group. In fact, we have documented that MSNA restrains the reflex muscle vasodilatation during exercise in patients with heart failure [44]. And, when the sympathetic activity was blockade with intra-arterial infusion of phentolamine (alpha-adrenergic antagonist), the increase in FBF and FVC was significantly improved [44]. Thus, we can speculate that in Gln27Gln group the exacerbated MSNA response during exercise could restrain the amelioration in vasodilation in these patients during our experimental protocol. It is possible to suggest that although Gln27Gln group match their sympathetic activity with the Gln27Glu+Glu27Glu group at the end of exercise training protocol, they may require a more prolonged period of exercise to improve vascular function and reduce oxidative stress. However, this hypothesis needs to be tested.

### Limitations

Our study has limitations. The frequency of patients with ACS homozygous for the Glu allele in our sample was significantly reduced compared with that in patients homozygous for Gln27Gln. In fact, in the Gln27Glu+Glu27Glu group, only 5 had the homozygous Glu27Glu polymorphism. However, the present frequency of distribution of these ADRB2 polymorphisms is in line with that in other studies that demonstrated that the Gln27Gln polymorphism is more frequent in patients with myocardial infarction than in healthy control subjects [15,16]. To increase the power of our study, we analyzed the presence of the Glu27 allele including patients homozygous for Glu27Glu and heterozygous for Gln27Glu in the same group in comparison with patients homozygous for the Gln27 allele.

### Perspectives

The higher MSNA and mean BP responses during exercise are suggestive of high risk in patients with ACS and the ADRB2 Gln27Gln polymorphism. Moreover, it brings about the idea that patients with the Gln27Gln polymorphism deserve special attention in clinical practice. Finally, exercise training should be strongly recommended to these patients.
